# Structural Manipulation of 3D Graphene-Based Macrostructures for Water Purification

**DOI:** 10.3390/gels8100622

**Published:** 2022-09-29

**Authors:** Zijun Yu, Li Wei, Lun Lu, Yi Shen, Yang Zhang, Jun Wang, Xiaoyao Tan

**Affiliations:** 1 State Key Laboratory of Separation Membranes and Membrane Processes, School of Environmental Science and Engineering, Tiangong University, Tianjin 300387, China; 2 School of Textile Science and Technology, Tiangong University, Tianjin 300387, China; 3 Department of Chemical Engineering, Tiangong University, Tianjin 300387, China; 4 State Environmental Protection Key Laboratory of Environmental Pollution Health Risk Assessment, South China Institute of Environmental Sciences, Ministry of Ecology and Environment, Guangzhou 510655, China; 5 Key Laboratory of Microbial Technology for Industrial Pollution Control of Zhejiang Province, College of Environment, Zhejiang University of Technology, Hangzhou 310032, China

**Keywords:** 3D graphene, structural manipulation, water purification, adsorption, catalysis

## Abstract

The rapid development of graphene-based nanotechnologies in recent years has drawn extensive attention in environmental applications, especially for water treatment. Three-dimensional graphene-based macrostructures (GBMs) have been considered to be promising materials for practical water purification due to their well-defined porous structure and integrated morphology, and displayed outstanding performance in pollutant abatement with easy recyclability. Three-dimensional GBMs could not only retain the intrinsic priorities of 2D graphene, but also emerge with extraordinary properties by structural manipulation, so rational design and construction of 3D GBMs with desirable microstructures are important to exploit their potential for water treatment. In this review, some important advances in surface modification (chemical doping, wettability, surface charge) and geometrical control (porous structure, oriented arrangement, shape and density) with respect to 3D GBMs have been described, while their applications in water purification including adsorption (organic pollutants, heavy metal ions), catalysis (photocatalysis, Fenton-like advanced oxidation) and capacitive desalination (CDI) are detailly discussed. Finally, future challenges and prospective for 3D GBMs in water purification are proposed.

## 1. Introduction

Graphene is a 2D sp^2^-hybridized carbon nanosheet with unique physical and chemical properties, including ultrahigh surface area (~2630 m^2^/g), outstanding carrier mobility (~10,000 cm^2^ V^−1^ s^−1^), high mechanical strength (Young’s modulus of ~1 TPa) as well as strong chemical stability in corrosive agents (acidic/alkaline solutions, organic solvents), and has demonstrated its effectiveness in versatile applications such as energy storage, electronics, biomedicine and environmental remediation [1,2]. Due to their open-layered network and adjustable structures, graphene and its derivatives are widely recognized as efficient adsorbents for aqueous organic and inorganic contaminants [3,4]. Moreover, graphene-based nanomaterials (GBNs) have displayed excellent performance in heterogeneous photocatalysis and Fenton-like reaction for pollutant elimination, which could serve as promising platforms for advanced water treatment [5,6,7].

Nevertheless, direct applications of GBNs in water treatment are unrealistic based on two aspects. On the one hand, graphene nanosheets are extremely easy to agglomerate due to the strong π-π interaction between the adjacent layers; on the other hand, GBNs at the nanoscale are difficult to recycle and may pose potential hazardous effects to human beings once released to the environment [8,9]. Notably, assembly of GBNs into 3D macroscopic devices is a smart approach to overcome these shortcomings. Owing to the hierarchical porous structure and integrated appearance, 3D GBMs could not only guarantee safety during applications, but also emerge with additional superiorities, e.g., fast mass transfer and easy manipulation, which hold great potential for practical applications [10]. Generally, graphene oxide (GO) is preferred over graphene for the “bottom-up” assembly of 3D GBMs, since the amphiphilic nature endows GO ease of processing in aqueous solution and avoids the irreversible agglomeration. Moreover, 2D GO nanosheets are mass-producible from natural graphite flakes via chemical oxidation followed by exfoliation, offering a practical route to 3D GBMs fabrication [11].

**Figure 1:**
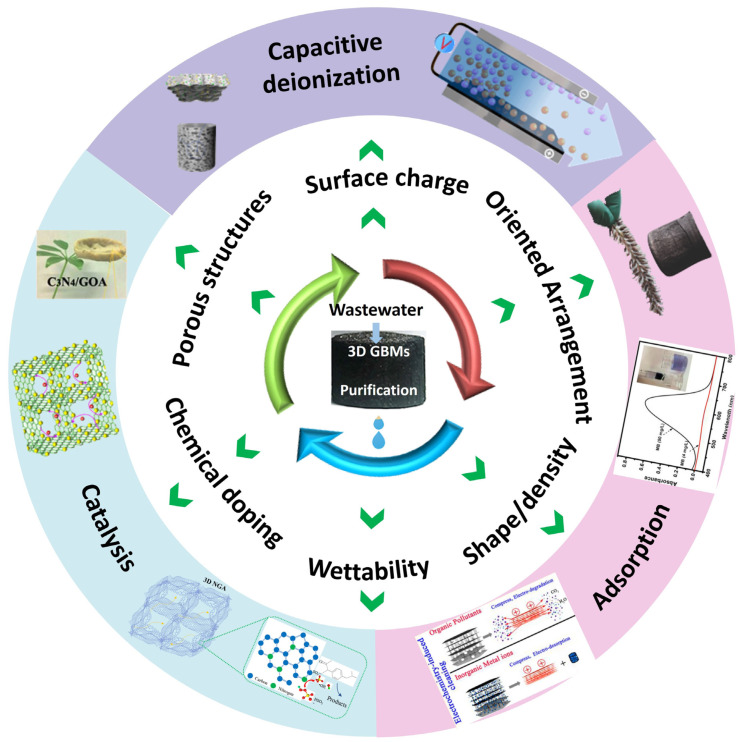
Schematic illustration of the structural manipulation of 3D GBMs for water purification applications.

In addition, multifunctional 3D GBMs can be fabricated through surface modification, geometrical control or hybridization to meet the demands of diverse applications [12]. For instance, heteroatoms doping or coassembly with metal/metal oxide nanomaterials could remarkably boost the activity in persulfate-based advanced oxidation processes (PS-AOPs) for pollutant removal, while the activity would be further enhanced by pore regulation [13,14]. Several recent reviews have summarized the preparation methods and composition control of 3D GBMs [15,16]; this critical review mainly describes the surface modification and geometrical control of 3D GBMs and further highlights their applications in adsorptive and catalytic removal of water contaminants (Figure 1). At the end, prospective developments and challenges for water purification are discussed.

## 2. Surface Modification

### 2.1. Chemical Doping

Chemical doping with substituent heteroatoms into the graphene lattice has been demonstrated to be a feasible strategy to break the chemical inertness of graphene and modulate its electronic and chemical features [17]. It was reported that the introduction of N (or B, P) atoms could form a delocalized conjugated system within the planar sp^2^-hybridized structure, which would create more active sites and dramatically enhance the chemical activity of graphene [18,19]. For example, N-doped graphene showed excellent electrocatalytic activity toward the oxidative dehydrogenation reaction (ODH) and oxygen reduction reaction (ORR); moreover, nitrogen modification of graphene gave rise to an astonishing improvement in peroxymonosulfate (PMS) activation for degradation of antibiotics and phenols [20,21]. In recent years, controlled assembly of the graphene into 3D macrostructures coupled with heteroatoms doping was expected to further expand the structural features of graphene-based materials while enriching their practical applications [22,23]. Therefore, tremendous efforts have been made to rationally manipulate the dopant types, doping level as well as codoping on 3D graphene-based macrostructures (GBMs) for various applications.

Template-directed CVD growth is an effective strategy to fabricate 3D graphene foams (GFs). Using a modified CVD method, Xue et al. prepared a class of GFs doped with nitrogen, boron or both [24]. Specifically, a nickel foam with 3D porous structure was employed as a scaffold for graphene growth, and N-doped GFs with a doping level of 3.1 at% were obtained by CVD of CH_4_ in the presence of NH_3_ at 1000 °C under Ar protection, followed by PMMA and HCl treatments to remove the nickel template. To obtain B-doped GFs, toluene and triethyl borate were used as a carbon or boron source during the CVD process, while a boron doping level of 2.1 at% could be reached. Additionally, B- and N-codoped GFs were prepared by CVD using melamine diborate as the precursor, and the doping level of nitrogen and boron were 4.5 at% and 3 at%, respectively. Notably, the resultant doped GFs showed much higher electrocatalytic activities toward ORR than their undoped counterparts; among them, B- and N-codoped GFs exhibited the highest reactivity, which should be attributed to the synergistic effect of B and N atoms that reduce the bandgap energy as well as increase the electroactive surface area. Furthermore, S- and N-codoped 3D graphene could be fabricated by CVD of C_2_H_4_ in the presence of NH_3_ using MgSO_4_-containing whiskers both as templates and sulphur source, whereas the doping level of S and N were 5.2 at% and 1.8 at%, respectively, thus resulting in a significant enhancement in conductivity and electrochemical performance [25].

**Figure 2:**
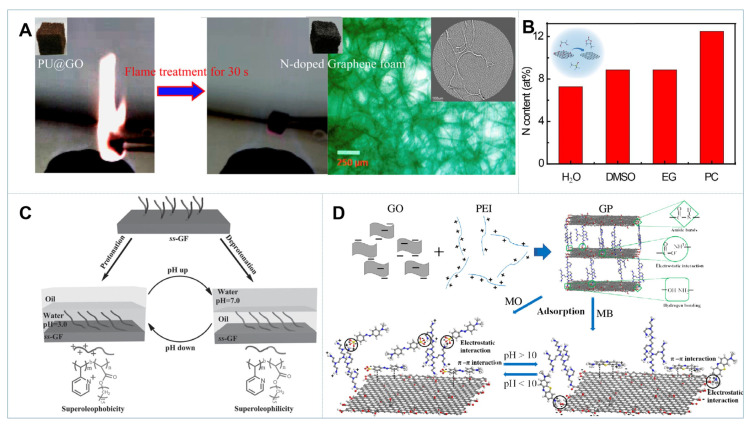
(**A**) Procedure for preparing N-doped graphene foam; [26] (**B**) N contents in the N-rGO organogels prepared in different solvents; [31] (**C**) Schematic illustration of switchable wettability of the as-synthesized ss-GF; [46] (**D**) Adsorption mechanisms of 3D GO/PEI aerogel with MB and MO under different pH values [49].

In addition to the CVD approach with complicated procedures, a simple and effective method was developed to fabricate NGFs through pyrolyzing a commercial polyurethane (PU) sponge coated with GO in an ethanol flame [26]. As shown in Figure 2A, the high temperature (~ 550 °C) of the flame could completely remove the PU template in less than 60 s in an open environment, resulting in the reduction of GO and simultaneous nitrogen doping of graphene layers, which was obviously faster than other approaches to prepare GFs with different hard templates. The NGFs inherited the highly porous open-cell structure of PU sponge with a remarkable 98.7% pore volume, which afforded a high absorption efficiency toward oil and organic solvents. Moreover, the N-doping level of NGFs (~8.3 at%) was much higher than those foams obtained through CVD growth (~3.1–4.5 at%), thereby leading to superior capacitive performance. Likewise, melamine spongy has also demonstrated to be effective as a template for 3D NGFs fabrication, while the post-treatment toward the GO-coated template has a big impact on the doping level of NGFs. Jiang et al. prepared graphene melamine foam and then annealed it at 1000 °C in the tube furnace under an Ar/H_2_ (9:1) atmosphere for one hour [27]. It was found that the N-doping level in the obtained NGFs was 1.82 at%, which was much lower than those foams acquired by microwave irradiation (5.7 at%) at 700 W for 5 s.

The assembly of chemically modified graphene (CMG) materials into functional gels is another facile strategy to fabricate 3D GBMs. Normally, the hydrothermal treatment of GO with a certain concentration can induce the self-assembly of graphene nanosheets into 3D hydrogels, while chemical doping could be easily achieved either by adding different doping precursors in the hydrothermal process or post-treatment of GFs in a special atmosphere (NH_3_, H_2_S) under high temperatures [13,22,28]. Nitrogen doping with ammonia resulted in the formation of 3D N-doped graphene aerogel (NGA) with high nitrogen content (8.4 at%) and excellent electrical conductivity and wettability, which exhibited a high CO_2_ adsorption capacity and electrochemical performance [29]. The organic amines, such as urea, ethylenediamine and dopamine, can act both as the nitrogen source and cross-linking unit to control the N-doping level, porous structure and density of 3D GBMs [30]. Particularly, organic media are favorable for improving the doping level of N-rGO through solvothermal reaction. Figure 2B illustrates that the N content of the freeze-dried organogel with propylene carbonate was as high as 14.01 at%, which exhibited a high conductivity of 182.4 ± 12.0 S m^−1^ [31].

Substitutional doping of 3D GBMs with other heteroatoms such as B, S and P can also tailor the charge distribution of graphene network, which could be easily realized by a hydrothermal treatment of GO and heteroatom precursors such as H_3_BO_3_, PEDOT:PSS and H_3_PO_4_ [32,33]. Beyond that, codoping with two elements with different electronegativities may give rise to a unique electron distribution, thus exhibiting a synergistic effect. Wu et al. synthesized boron- and nitrogen-codoped graphene aerogel (BN-GAs) using GO and NH_3_BF_3_ as precursors via the hydrothermal method and freeze-drying process [34]. It was revealed that significant contents of nitrogen (≈3.0 at%) and boron (≈0.6 at%) were incorporated in BN-GAs. Compared with NGA and BGA, BN-GAs showed higher specific capacitance, rate capability and energy density, suggesting the effectiveness of codoping with two elements. Likewise, other precursors such as NH_4_SCN, NH_4_B_5_O_8_ and melamine phosphate could be employed as codoping precursors [35,36,37]. Notably, it was reported that trace boron (0.1 wt%) apparently promoted PMS activation by B- and N-doped graphene, while an inferior activity was attained when the boron content was improved to 0.25 wt%, suggesting that the codoping contents and species of 3D GBMs should be well-controlled to exploit the synergetic effect [38].

### 2.2. Wettability

The wettability of 3D GBMs plays an important role in governing the interactions between graphene and water, which also have a significant impact on the adhesion and electronic properties of 3D GBMs [39]. The wettability can be quantified by measuring the contact angle (CA) of a water droplet placed on the surface of a solid. The surface is defined as hydrophobic when its CA is higher than 90°; otherwise, it is hydrophilic. Once the CA is greater than 150°, the surface of 3D GBMs is termed as superhydrophobic, which means it is extremely water-repellant and favorable for various applications, e.g., oil/water separation and self-cleaning surfaces [40].

Taking inspiration from natural studies that have exploited surface roughness and low-surface-energy coatings to engineer superhydrophobic surfaces, multifarious superhydrophobic 3D GBMs have been fabricated with outstanding water repelling ability and oil absorption performance. Dong and coworkers have devised a two-step CVD method to prepare superhydrophobic 3D graphene–CNT hybrid foam [41]. Due to the nanoroughness created by the CNT forest grown on the graphene surface, the hybrid foam exhibited superhydrophobic properties with a CA of 152.3°. It could selectively remove oils and organic solvents from water with high capacity and good recyclability. The surface roughness-induced superhydrophobicity of 3D GBMs could also be attained via the incorporation of carbon black, SiO_2_ nanoparticles and many other nanomaterials [42,43]. Beyond that, a variety of low-surface energy coatings such as Teflon, thiols and fluorinated silanes could conveniently be attached to the graphene foam to impart the superhydrophobicity to the surface [44].

It is commonly believed that 3D GBMs have remarkably higher affinity to organic solvents than water-soluble contaminants due to their high hydrophobic surface. To meet the requirements for various wastewater treatment, a rational design of 3D GBMs with switchable wettability is highly desirable. Chang et al. fabricated a bulk 3D GBM with switchable hydrophilicity/hydrophobicity by O_3_ or annealing treatment to tune the surface chemical composition [45]. The resulting GBMs displayed excellent and reversible absorption performances to a variety of dyes and organic liquids, with a broad water CA range from 41.3° ± 3.2° to 118° ± 1.8°. In another study, Zhu et al. modified 3D porous GH with a smart pH-responsive surface by grafting a block copolymer (P2VP-b-PHA) [46]. Figure 2C depicted that the as-designed smart GF exhibited superhydrophilicity or superhydrophobicity in the acidic or neutral medium, respectively. It not only showed superior absorption capacity for oil and organic molecules, but also had good recyclability due to the fast reversion of organic adsorption and desorption by varying the medium pH. Furthermore, by coassembly with hydrophilic molecules such as poly(vinyl alcohol) and phytic acid, the resultant GBMs showed superamphiphilic nature, thereby manifesting high affinity for both polar water pollutants and nonpolar organic liquids [47,48].

### 2.3. Surface Charge

Given that 3D GBMs derived from GO were generally negatively charged, to effectively abate charged pollutants, the fabrication of surface charge tunable GBMs has been demonstrated. As presented in Figure 2D, Zhao et al. associated positively charged poly(ethyleneimine) (PEI) with GO to fabricate 3D GO/PEI (GP) aerogels [49]. Compared with the pristine GO aerogel which was negatively charged throughout the pH range (2.0–10.5), surface charges of the obtained GP aerogels could be well-controlled via changing solution pH with an isoelectric point (IEP) of ~10. So, GP was capable to selectively remove cationic and anionic dyes in different aqueous conditions. Moreover, the surface charge of 3D GBMs can also be facilely regulated by coassembly with some other polycations, such as polylysine (PLL) and polydimethyldiallylammonium chloride (PDDA), or grafting amine functional groups on the surface using 3-aminopropyltriethoxysilane (APTES). Surface modification provided an optimized interface for adsorptive and catalytic removal of versatile charged pollutants in water [50,51,52].

## 3. Geometrical Control

### 3.1. Porous Structure

3D GBMs possess hierarchical porous structures. Among them, in-plane micropores (<2 nm) and wrinkled mesopores (2–50 nm) provide high surface area, while macropores (>50 nm) guarantee accessibility to the surface. Pore size is a critical structural parameter of 3D GBMs, and the appropriate sizes of pores in 3D GBMs can exploit potential specific applications such as adsorption/separation, capacitive deionization (CDI) and heterogeneous catalysis [5,53]. Self-assembly of 3D GBMs through gelation is an important strategy to produce well-defined and interconnected porous network. The microstructure could be well-controlled by changing the GO concentration, sheet size, solution pH, drying conditions and other parameters. Moreover, through pre-/post-treatment (H_2_O_2_ etching, chemical activation, etc.) or combination with soft and hard templates, it could offer a high level of control over the hierarchical porous structure of 3D GBMs.

In 2010, Xu et al. first demonstrated a one-step hydrothermal process to self-assembly of 2D graphene sheets into 3D macrostructures. Due to the cross-linking through coalescing or partial overlapping of the flexible graphene nanosheets, as low as 1 mg/mL of GO dispersion could form a stable hydrogel network after 12 h hydrothermal reduction at 180 °C. Their pore sizes ranged from sub-micrometer to several micrometers [54]. By combination of oxalic acid (OA) and sodium iodide (NaI) as reducing agents, Zhang et al. developed a facile reduction-assembly strategy to prepare 3D graphene assemblies from GO suspension with ultralow concentrations (0.1 mg/mL) [55]. The as-obtained 3D network was composed of massive interconnected macropores, while the pore sizes decreased from 24.2 ± 3.5 μm to 2.1 ± 0.4 μm with increasing GO concentration (0.1–4.5 mg/mL). Furthermore, the pore size of 3D GBMs could also be effectively regulated by altering the lateral sizes of GO via hydrothermal self-assembly [56]. It was found that the pore size of the 3D GF was positively correlated with the lateral size of GO, and the smallest average size was ~500 nm. Notably, the surface area and total pore volume of the resultant GF increased with decreasing the lateral size of GO, possibly attributed to the fact that the smaller GO sheets were more effective in avoiding the folding and restacking problems. Researchers have also demonstrated that solution pH of GO dispersion had a profound impact on the graphene morphology and subsequently affected the porous structure of 3D GBMs [57]. The graphene nanosheets were bent and twisted under pH 11 after hydrothermal assembly, which reduced layer stacking and connected each other through the bent borders, hence leading to more open structures and higher pore volumes. In contrast, the flatter nanosheets attained at pH 3.0 entailed more stacking between the basal planes finally resulted in less porous and denser aerogels.

**Figure 3:**
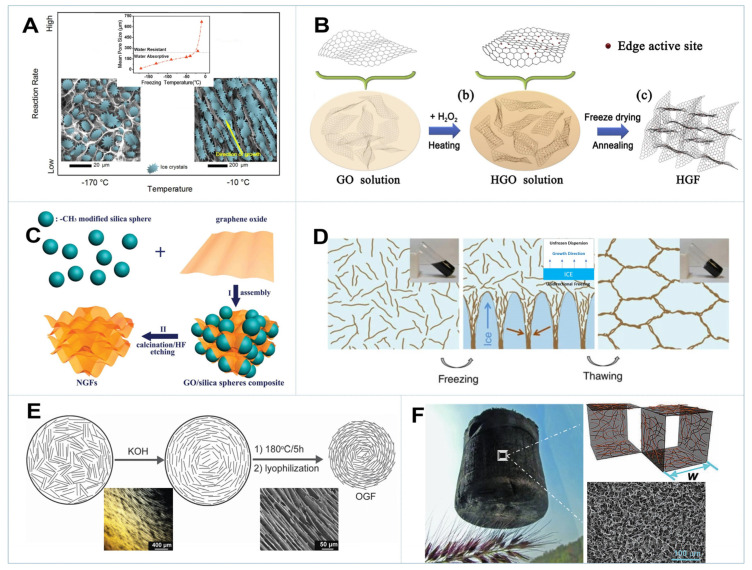
(**A**) The pore size of graphene aerogel as a function of freezing temperature [58]. (**B**) Synthesis procedures of holey graphene framework (HGF) [64]. (**C**) Schematic illustration of the fabrication processes of the nanoporous graphene forms (NGFs) [70]. (**D**) Schematic depicting the formation of oriented graphene monolith by freeze casting [84]. (**E**) Fabrication of oriented graphene foams (OGFs) by base-induced method [88]. (**F**) An ultraflyweight carbon aerogels fabricated by coassembly of graphene and carbon nanotubes [97].

Despite simply altering the hydrothermal parameters, Xie et al. reported a freeze-casting strategy for large-range tailoring the porous structures and properties of graphene foam by varying the freezing temperature [58]. As shown in Figure 3A, with the increase in freezing temperature from −170 °C to −10 °C, the pore morphology of 3D GAs changed substantially from uniform and cellular to anisotropic and lamellar structures, while the pore sizes and wall thicknesses increased by 80 times (10–800 μm) and 4000 times (20 nm−80 μm), respectively. Such a significant variation should be attributed to the change in freezing rate, which decides the crystal shape and affects the morphology of microcells in the aerogels. A large number of mesopores could be fused together to generate macropores, impelled by the large ice crystals growing at high freezing temperatures [58,59]. Ice crystals in fact serve as templates for the formation of the porous structure of graphene aerogel, and their microstructure, porosity, orientation and properties could be finely tuned by controlling the freezing rate and direction [60,61]. Other than freeze-drying, supercritical CO_2_-drying offers an effective strategy to form an aerogel from a hydrogel precursor while preserving its original network. Under supercritical conditions, there is no gas/liquid interface in the system, the associated capillary forces are eliminated and the fluid can be removed, leaving the network intact and relatively uncompressed [59,62]. It was unveiled that the supercritical CO_2_-dried graphene aerogel showed a much higher pore volume than the freeze-dried sample (2.48 vs. 0.04 cm^3^ g^−1^), demonstrating the effectiveness of supercritical drying in producing hierarchical porous aerogel [59]. Air-drying or heat-drying generally lead to the shrinkage of graphene aerogel. Due to the strong capillary force generated by the evaporation of trapped water in the hydrogel, the interconnected macropores were compressed into highly compacted stackings and posed a low surface area and porosity [63].

Considering that in-plane micro-/mesopores are rich in edge defects and can provide more active sites and efficient transport pathways for both adsorption and heterogeneous catalysis, Li et al. reported the construction of 3D holey graphene framework (HGF) by hydrothermally reduction of GO in the presence of H_2_O_2_ (Figure 3B) [64]. During this process, the rGO nanosheets tended to self-assembly into the interconnected framework, while H_2_O_2_ preferentially etched the defective sites on the graphene sheets to generate abundant in-plane nanopores. By varying the concentration of H_2_O_2_, the etching degree can be tuned, producing HGFs with various degrees of porosity, which had considerable potential for CDI desalination applications and beyond [64,65]. Likewise, the creation of in-plane pores could also be achieved by chemical or physical activation with the addition of H_3_PO_4_, KOH, steam, etc. [66,67,68]. There was a huge improvement in pore volume in 3D GBMs after activation, with much of the pores distributed in the range of 0.8–10 nm depending on the activation method, temperature and activator concentration, as summarized in Table 1. Moreover, Lacey et al. combined thermal air oxidation with liquid-phase oxidation to prepare nanoporous GO with 4–25 nm through-holes, which could serve as ink for the 3D printing of hierarchically porous graphene framework to promote full active-site utilization [69].

Another important method to fabricate 3D GBMs with controllable porosity involves the introduction of hard or soft templates. As shown in Figure 3C, methyl-group-grafted silica spheres were chosen as a hard template and mixed with GO to coassemble into a 3D framework [70]. After calcination under argon atmosphere and silica etching by HF, nanoporous graphene foams (NGFs) with controllable pore size (30–120 nm) could be obtained by simply tuning the particle size of silica spheres, which also exhibited ultrahigh pore volumes (~4.3 cm^3^ g^−1^) and surface areas (851 m^2^ g^−1^). Similarly, some other metal or metal oxide nanoparticles such as Co_3_O_4_, Fe_3_O_4_ and Ag could also be used as templates to regulate the interconnected mesopores on 3D GBMs [70,71,72]. Notably, the integration of GA with microporous ZIF-8 could manifest an excellent synergistic effect of hierarchical pore structures [73]. In addition to hard templates, polystyrene (PS) microspheres with different particle sizes (0.5–2 μm) could be employed as soft templates to fabricate 3D GA with diversified pore sizes [74]. After mixing with GO with vigorous agitation, uniformly distributed PS spheres were decorated between the graphene nanosheets, followed with freeze-drying and calcination to remove PS template, and a macroporous 3D GA with uniformly dispersed cavities could be acquired. Beyond that, template-directed CVD growth is also a widely used method to tune the hierarchical porosity of 3D GBMs, which has been detailly overviewed by a recent review [75].

**Table 1:** Comparison of various 3D GBMs in terms of their synthesize methods, porous structures and performance for different applications.

Carbon Source	Method	Pore Sizes	Pore Volume (cm^3^/g)	Surface Area (m^2^/g)	Density (mg/cm^3^)	Performance	Ref.
GO	Chemical reduction and Freeze-drying	1.5–55 nm	0.04	12	12–38	A specific capacity of 76 F g^−1^	[59]
GO	Chemical reduction and Supercritical CO_2_ drying	2–100 nm	2.48	512	23–96	A specific capacity of 128 F g^−1^	[59]
GO, pyrrole	Hydrothermal reduction and Heat drying	Center at 4 nm	0.28	236	-	A specific capacity of 163 F g^−1^	[63]
GO	Hydrothermal reduction (with different GO size)	0.5–10 μm	0.26–1.39	98–402	15–80	High adsorption capacity for MB (215 mg g^−1^)	[56]
GO	Hydrothermal reduction and H_2_O_2_ etching	2–4 nm	-	124	-	High desalination capacity (29.6 mg g^−1^)	[64]
GO, MA foam	KOH activation	0.8–13 nm	0.08–0.4	73–944	-	A high specific capacitance of up to 217 F g^−1^	[68]
GO	Hydrothermal reduction and Steam activation	Center at 2.5 nm	3.6	867	-	High adsorption capacity for CO_2_ (10.8 wt%)	[66]
GO, Urea	Hydrothermal reduction and H_3_PO_4_ activation	0.8–8 nm	0.25–0.81	473–1145	-	A specific capacity of 204 F g^−1^	[67]
GO, ZIF-8	Hydrothermal and layer- by-layer assembly	3–10 nm	0.4–0.67	404–1567	-	High CO_2_ uptake capacity (0.99 mmol g^−1^, 298 K)	[73]
GO	Template (emulsion and ice templating)	2–100 nm 7–60 μm	-	170–422	1–200	An ultrahigh absorption capacity for motor oil (605 g g^−1^)	[76]
GO	Hard template (SiO_2_)	30–120 nm	4.28	851	-	A reversible capacity of 750 mAg^−1^ in lithium ion batteries	[70]
GO	Soft template (SDS)	40–120 μm	-	-	~5–48	High compressive stress of 5.4 MPa	[77]
GO	Hard template (Ag)	Center at 5, 30, 100 nm	-	199–966	-	High output power density of 1.15 W m^−2^	[72]

### 3.2. Oriented Arrangement

As it is known, the macroscopic properties of 3D GBMs are strongly dependent on the pattern of how individual graphene nanosheets interconnect with each other [78]. The oriented arrangement of graphene nanosheets in 3D GBMs endows unique and anisotropic mechanical and electronic properties. It also provides barrier-free paths for the directional transfer process [79,80]. Nevertheless, since GO building blocks with structural heterogeneity usually induce disordered self-assembly, most of the reported 3D GBMs so far present random cross-linked structures, which severely restricts mass transport and leads to nonoptimal mechanical properties [81]. Herein, several effective strategies for the ordered assembly of 3D GBMs are summarized.

Zhu et al. proposed a direct ink writing (DIW) strategy for 3D printing of periodic graphene aerogel microlattices [82]. Through computer-aided design, printable GO ink was extruded through a micronozzle immersed in isooctane followed by supercritical drying and thermal annealing. Furthermore, 3D-printed GAs with periodic macroscale pores could be easily obtained, while the microstructures and properties of the microlattices could be tuned by changing the ink formulation. It is worth mentioning that the ink viscosity should be strictly controlled because a high viscosity is essential for the material to keep its shape on the printing bed. So, either high GO concentration or the addition of viscosifier are required to enhance the printability of the GO-based inks [83].

Freeze-casting is an important strategy to drive the oriented assembly of 3D GBMs based on a proper combination of graphene chemistry and ice physics. As shown in Figure 3D, in a common freeze-casting process, when graphene-based precursors were directly frozen in cold sources, ice crystals grew from the outside to the inside of the dispersion. Phase separation would cause the accumulation of graphene nanosheets between the ice crystals, therefore leading to a randomly oriented porous structure [58,84]. With unidirectional freezing, Qiu et al. have demonstrated that a highly ordered 3D GBM with microhoneycomb structure and superelasticity could be obtained, but freezing a GO dispersion without prereduction only yielded a disordered 3D porous structure [84]. It was suggested that the oxygen-containing groups on GO sheets preferably adsorbed on the surface of ice crystals in liquid water via H-bonding and resulted in a curved ice crystal surface. The partial removal of oxygen-containing groups on rGO reduced the constraints on the growth of the ice crystals, thus producing a 3D framework with vertically aligned structures. When the reduction degree was further increased, the aqueous rGO dispersions turned into a stiff hydrogel. It might not be possible to acquire the desired structure through freeze-casting [85]. Moreover, a GO aerogel with both vertically and radically aligned structure could be fabricated by adding 3 vol% of ethanol to the GO dispersion, followed by a bidirectional freeze-casting process (BDF) [83]. It was speculated that the addition of ethanol could decrease the H-bonding between GO and water, thus facilitating the radical growth of ice crystals. The resultant aerogel had high porosity with a specific oriented structure, which has shown superior elasticity and absorption capacity to organic solvents compared with conventional 3D GBMs. Beyond that, some other molecules such as PVA and chitosan could also act as structure-directing agents [61,86].

The liquid crystals (LC) behavior of GO has also afforded new solution-processing methods to the assembly of highly ordered GBMs [87]. However, the formation of nematic LC phase using normal-sized GO sheets (average lateral size < 10 μm) usually requires a high GO concentration (~10 mg/mL), which cannot be used to prepare oriented GFs with low density [88]. As depicted in Figure 3E, Yao et al. have demonstrated that strong bases such as KOH could induce the formation of GO LC with highly oriented microstructures at low concentration (3.5 mg/mL). A long-range ordered GF with low density and good elasticity can be obtained upon hydrothermal reduction of GO LC [88]. Moreover, the orientation of GO LC could be easily manipulated by solution-mixing or the surfactant-foaming strategy [81]. Inspired by the LC-assisted assembly, Deng et al. have reported a novel protocol for guiding the oriented arrangement of graphene sheets. Through the addition of electrolytes that break the electrostatic balance to form GO microclusters, subsequent vacuum filtration induces the shear force to direct the oriented assembly of GO sheets [89]. After freeze-drying and thermal reduction, ordered porous graphene materials (OPG) could be obtained. The oriented arrangement endows OPG with anisotropic mechanical, electrical and thermal properties, as well as excellent compressive and recovery capability, thus manifesting superior performance in organic pollutants removal and pressure sensing.

### 3.3. Shape and Density

To utilize GBM devices for specific applications, their macroscopic shape should be well-manipulated. Based on the hydrothermal assembly using GO as the precursor, it has been reported that the shape and dimension of the as-obtained graphene hydrogel largely depend on the reactor and the GO concentration. The resultant 3D graphene hydrogel can be cast into any desired shapes depending on the shape of reaction vessels since the shrinkage of the hydrogel during the self-assembly is isotropic [90]. By vacuum filtration of reduced graphene dispersions, an ordered graphene hydrogel (OGH) film with circular shape could be obtained. The thickness is linearly correlated with the volume of graphene dispersions filtered, which have shown good performance in nanofluidic generators, pressure sensing as well as pollutants interception [89,91]. After freeze-drying, the as-obtained aerogel film could be facilely cut into different appearances (rectangle, hexagon, triangle, etc.). Moreover, the macroscopic shape of 3D GBMs could also be effectively modulated by 3D printing and template-directed assembly approaches [92,93].

As discussed above, the microscopic structure of graphene sheets has a significant impact on the overall properties of 3D GBMs. Due to the face-to-face restacking of graphene substrates within the 3D framework, some graphene foams still suffer from the reducing surface area and limiting mass transfer process [65,66]. Recently, Fang et al. have proposed a facile mild-reduction methodology to induce GO nanosheets curling up into nanoscrolls, and self-assembly to fabricate graphene nanoscroll (GNS) foam [94]. Formamide (FA) could act as a bridge that cross-linked GO sheets into 3D network under mild heating, while the adsorbed FA molecules within the hydrogel would increase the surface stress of GO sheets and induce rolling up of GO sheets into nanoscrolls during the freeze-drying process. The formation of GNSs inside the 3D framework largely relieved the restacking of graphene sheets, which not only supplied more available sites for pollutant enrichment, but also facilitated the pollutants’ diffusion through an impeded interior network space, thereby paving a broader avenue for the applications of GNSs-based macrostructures in water purification and related fields [95].

Density is one of the most important parameters for 3D GBMs applications, which is closely related to the macroporous structure of aerogels [96]. As it is known, the density of the freeze-dried graphene aerogel made from GO solution is positively correlated with the GO concentration (C_GO_). If C_GO_ is low (e.g., <0.5 mg/mL), the 3D framework would collapse due to the lack of connection among graphene sheets [54,55]. By integrating the giant graphene cell walls with elastic CNT ribs via a “sol-cryo” protocol, Sun et al. have constructed ultraflyweight aerogels (UFAs) with a density as low as 0.16 mg cm^−3^ (Figure 3F) [97]. In the formation of UFAs, GO sheets were functioning like concrete blocks, while the interspersed CNTs were acting like the rebar to enhance the connection between GO sheets. The ideal combination of graphene and CNT offered the UFAs outstanding elasticity, ultralow density and extremely high absorption capacities for various solvents and oils [97,98]. As shown in Table 1, the densities of 3D GBMs obtained by GO assembly varied in the range of 1–200 mg cm^−3^. Furthermore, large-scale manipulation of the density of carbon-based aerogels (from 2.5 to 1327 mg cm^−3^) has been achieved by means of altering the type of carbon precursor (GO, CNTs, graphene nanoribbons), precursor concentration and post-treatment conditions (drying method, pyrolysis) [99]. This research provided in-depth understandings of the relationship between the aerogel density and synthesis conditions.

## 4. Water Purification Applications

### 4.1. Adsorption

#### 4.1.1. Organic Pollutants

Organic pollutants are widely distributed in natural waters and pose potential detrimental effect to the ecosystem and human health. Three-dimensional GBMs display excellent capability in pollutant management due to their large surface area, well-defined porous structure and integrated morphology, which facilitate the mass transport and enrichment of organic pollutants with easy recyclability [16]. As presented in Table 2, 3D GBMs exhibit ultrahigh adsorption performance for various organic pollutants, including dyes, antibiotics, phenolic compounds and other emerging contaminants, which might be even higher than some common nanomaterials such as biochar, carbon nanotubes, montmorillonite, activated carbon, etc. [100]. Nonetheless, their adsorption capacities toward different pollutants range from tens to thousands mg g^−1^, which could be greatly impacted by the surface properties, porous structures as well as solution conditions [101].

Single-layered GO solution has demonstrated high efficiency and broad applicability in water purification; however, its practical application is restricted due to the difficulty in separation and recovery as well as the potential health risks arising from its nanotoxic effect. Liu et al. developed a 3D GO sponge through centrifugal vacuum evaporation using GO suspension (10 mg mL^−1^) as the precursor, displaying fast and efficient adsorption for MV (*Q*_max_ = 467 mg g^−1^) and MB (*Q*_max_ = 397 mg g^−1^) dye with equilibrium attained in 2 min, which could also be quickly recovered by vacuum filtration [101]. GO sponge inherited the intrinsic superiority of GO nanosheet while combining the integrated porous framework, thereby guarantee the strong surface interaction, fast diffusion and ease of regeneration. As it is known, the gelation of GO is closely related to its concentration, and the critical gel concentration that GO can self-generate usually reaches 10–30 mg mL^−1^. Such high values will give rise to the restacking and agglomeration of GO, which are disadvantageous for their environmental applications. Therefore, multiple cross-linking agents such as polymers, small organic molecules, multivalent ions and nanomaterials have been applied to drive the self-assembly of 3D GBMs [108].

Fang et al. fabricated 3D GO aerogels with nanoscaled Mg-Al LDHs as a cross-linker. Due to the cation-π interactions and charge-assisted hydrogen bonds, stable hydrogels could be formed with GO concentration as low as 2.5 mg mL^−1^. The obtained LDH+GO aerogels possessed more available sites and better structural stability than neat GO aerogels, which manifested high performance in dye removal from water [109]. By using biopolymers including DNA, BSA and chitosan (CS) as cross-linkers, a series of 3D GO gels were prepared and manifested ultrahigh adsorption capacities for cationic MV (1350 mg g^−1^) and MB (1100 mg g^−1^) dyes [49,108,109,110]. Notwithstanding, GO-biopolymer gels presented a much lower adsorption capacity for anionic and nonionic pollutants (CR, RhB, BPA, p-NP). It was possibly ascribed to the negatively charged surface with abundant O-containing groups that limited the performance for pollutant abatement [111].

**Figure 4:**
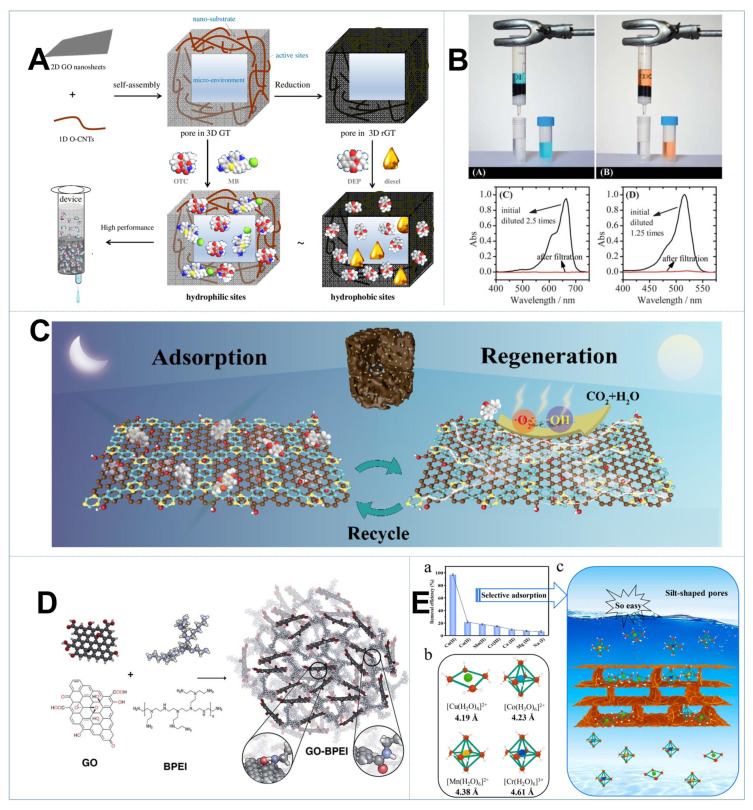
(**A**) The synergistic effect of GO and CNTs in 3D GTs’ structure and adsorption performance [98]. (**B**) Removal of MB and Eosin Y from water by filtration [110]. (**C**) Adsorption of trace organic micropollutants and regeneration under sunlight by 3D GO-CTF aerogel [116]. (**D**) Fabrication of 3D GO-BPEI aerogel [119]. (**E**) Schematic illustration of selective removal of Cu(II) by Sr-G/M [126].

CNTs are another kind of fascinating carbon nanomaterials with superior physicochemical properties and can be assembled into 3D macrostructures. By coassembly of 1D CNTs and 2D GO via a lyophilization strategy, 3D GT aerogels could be constructed with extremely low density and high stability. The minimum GO concentration required to form stable aerogel was even reduced to 0.2 mg mL^−1^ [98]. As shown in Figure 4A, due to the synergistic effects of CNTs and GO that ensured larger interspace and more exposed sites, 3D GT aerogels demonstrated high performance in organic adsorption. What’s more, the microenvironment of 3D GT aerogels could also be purposely regulated to achieve selective elimination of particular contaminants. Pore size is known as a key parameter that determines the properties and potential applications of 3D GBMs. Deng et al. prepared graphene foams (GFs) with controllable pore sizes by hydrothermal reduction of GO sheets with different lateral sizes [56]. It was found that S-GF assembled from small GO sheets had a much smaller mean pore diameter of ~500 nm than that of M-GF (2–5 μm) and L-GF (~10 μm). S-GF preserved higher SSA due to the fewer restacked sheets, and displayed the highest adsorbed amount of MB over the three GFs. In contrast, L-GFs had lower density and manifested higher absorption capacity for oil. These findings implied that large pores facilitated oil absorption and mass transport, while small ones were beneficial for aromatic compounds adsorption. That is to say, rationally designing the pore structure of 3D GBMs is highly desirable to fit the scope of different applications. Additionally, fabrication of 3D GBMs with highly oriented porous structure or nanoscrolls could further decrease the diffusion resistance and provide more active sites, which are favorable for adsorption and catalysis [89,94].

Having benefited from the 3D interconnected framework with intact structure and good biocompatibility, GA was able to efficiently enrich biogenic amines from soy sauce and histamine from red wine, making it very promising in practical applications [112,113]. As presented in Figure 4B, [110] 3D GBMs could serve as column packing to construct an adsorption column for practical water purification, which was driven by gravity without energy input and allowed nonstop operation. Fabrication of graphene-based hydrogel membrane is another smart approach to efficiently intercept organic pollutants with high water flux [89,114]. To achieve a sustainable purification process, conventional regeneration strategies including chemical desorption and thermal treatment in steam are effective but time-consuming and costly. Pan et al. demonstrated that GA could achieve in situ regeneration via electrochemical desorption/degradation without adding any other chemicals since GA had ultrahigh conductivity as well as electrochemical activity [115]. As shown in Figure 4C, by coupling graphene nanosheets and covalent triazine frameworks via freeze-drying, adsorption–photocatalytic dual-functional aerogels could be acquired. They exhibited excellent adsorption capability and self-regeneration performance for organic pollutants, paving a promising way to control organic micropollutants [116]. As will be discussed in the next section, 3D GBMs have been reported to efficiently activate persulfate and hydrogen peroxide for rapid degradation of organic pollutants. It is expected that the saturated adsorbent could be well-regenerated by mixing with these oxidants for advanced oxidation, which deserves further in-depth investigation.

#### 4.1.2. Heavy Metal Ions

Heavy metal ions are another kind of contaminant that is ubiquitous in the aqueous environment with direct hazardous effects on human beings [117]. Various purification technologies including membrane filtration, biological treatment, coagulation, chemical precipitation and adsorption, etc., have been applied for the elimination and recovery of metal ions from wastewater [118]. Among them, adsorption is an economical and efficient approach for heavy metal purification. It has been demonstrated that GO nanosheets with abundant oxygen-containing groups and large surface area exhibited ultrahigh adsorption capacity toward metal cations due to the strong electrostatic interaction as well as surface complexation. The assembly of GO into 3D GBMs not only could inherit the superiority of GO in metal ion enrichment, but they are also easy to recovery and recycle, thus affording effective platforms for water purification [95].

Generally, 3D GO monoliths acquired by freeze-drying of GO suspension were fragile and prone to disintegrate in water, so small molecules or nanomaterials were coassembled with GO to increase the structural integrity and active sites [95]. Fang et al. reported the fabrication of 3D GO monoliths based on the cross-linkage of small organic molecules containing double hydroxyl/carboxyl groups. It was unveiled that hydroxyl-containing agents were more conducive than carboxyl-containing molecules to induce the self-assembly of 3D GO with considerable stability and plasticity. What’s more, the GO monoliths cross-linked by glycol containing double hydroxyls manifested superior adsorption capacity toward Pb^2+^ removal (101.1 mg g^−1^) with good recyclability, probably as a result of the generation of more dissociable groups for enhanced Pb^2+^ binding [95]. Other than small organic molecules, several macromolecules and nanomaterials such as β-cyclodextrin (β-CD), chitosan (CS), polyethyleneimine (PEI), sodium alginate (SA), layered double hydroxides (LDHs) and MnO_2_ were introduced for the functionalization of 3D GO, which exhibited excellent performances in adsorptive removal of various heavy metal ions, as presented in Table 2 [105,110,119,120,121].

It was revealed that the valence, ionic radius and hydration energy of metal ions were the main factors affecting the affinity between heavy metal and 3D GBMs [105]. As illustrated in Figure 4D, Pakalski et al. constructed branched PEI modified GO foams (GO-BPEI) with affluent amine groups for sequestering heavy metal ions. The maximum adsorption capacities for Pb^2+^, Cd^2+^ and Cu^2+^ were 3390, 2051 and 1096 mg g^−1^, respectively, which was attained at pH = 5.0 and outperformed other typical sorbents such as biochar and activated carbon [119]. With the same valence, Pb^2+^ with lower hydration energy (1481 kJ mol^−1^) than Cd^2+^ (1807 kJ mol^−1^) and Cu^2+^ (2100 kJ mol^−1^) was more easily separated from the solution and exchanged with the functional groups on GO-BPEI, thus showing the highest adsorption affinity. For anionic toxic metals such as Cr(VI), As(V) and U(VI), the combination of polycations with negatively charged GO was essential to increase the surface charge and active sites of 3D GBMs [122,123,124]. By coassembly of GO and CS, the GO-CS aerogel exhibited high adsorption capacities toward U(VI) over a broad pH range, specifically, at 200, 319.9 and 384.6 mg g^−1^ at pH 3.5, 5.0 and 8.3, respectively [103]. Extended X-ray absorption fine structure spectroscopy (EXAFS) unraveled that the strong U(VI) adsorption was attributed to the inner-sphere surface complexation with –COO^−^, –OH and –NH_2_ groups, while the –NH_2_ groups were more favorable for U(VI) binding at mild alkaline pH [103].

As it is known, the interconnected porous network of 3D GBMs is beneficial for the transport and diffusion of heavy metal ions, while the adsorption rate is related to the intraparticle and external diffusion [125]. It was uncovered that the adsorption of Cu^2+^ on GO aerogel experienced a fast and slow adsorption stage before equilibrium was reached at 15 min. The adsorption rate was higher at the beginning due to the large surface area available for metal ions adsorption, but it slowed down as the reaction proceeded. It was possibly ascribed to the exhaustion of external sites that enforced intraparticle diffusion of adsorbate into interior sites [124]. Notably, as shown in Figure 4E, Hao et al. developed a 3D graphene oxide/montmorillonite composite aerogel (named Sr-G/M) with a controllable porous structure, and Sr-G/M was successfully selectively removed Cu^2+^ (97.1%) in intricate wastewater systems, which were also easily processed with excellent reusability, confirming the critical role of porous structure in pollutant abatement [126]. Moreover, the interlayer gaps were also instrumental in transmitting small molecules during adsorption, which could be modulated by introducing nanomaterials in 3D GBMs. For instance, manganese oxides (δ-MnO_2_) were used to deposit on the GO framework, endowing the adsorption of heavy metal ions either on the surface or intercalate interlayer gaps, thereby displaying outstanding adsorption capacity and fast kinetics toward various heavy metals [105,126].

### 4.2. Catalysis

#### 4.2.1. Photocatalysis

Photocatalysis is an environmentally friendly and cost-effective strategy for water treatment. Plenty of metal and metal-free semiconductor photocatalysts have been developed for efficient photocatalytic oxidation or reduction of environmental pollutants [15]. Despite the impressive advantages, conventional catalyst nanoparticles in powder form are easily agglomerated and hard to recycle, which largely restrict their environmental applications. Benefited from the superiorities of 3D GBMs, a series of semiconductors such as TiO_2_, ZnO, Bi_2_WO_6_, AgBr and g-C_3_N_4_ have been hybridized with 3D GBMs. These hybrid materials displayed remarkably enhanced photocatalytic performances in pollutant abatement, holding vast potential for practical applications [127,128,129,130,131,132,133,134].

**Figure 5:**
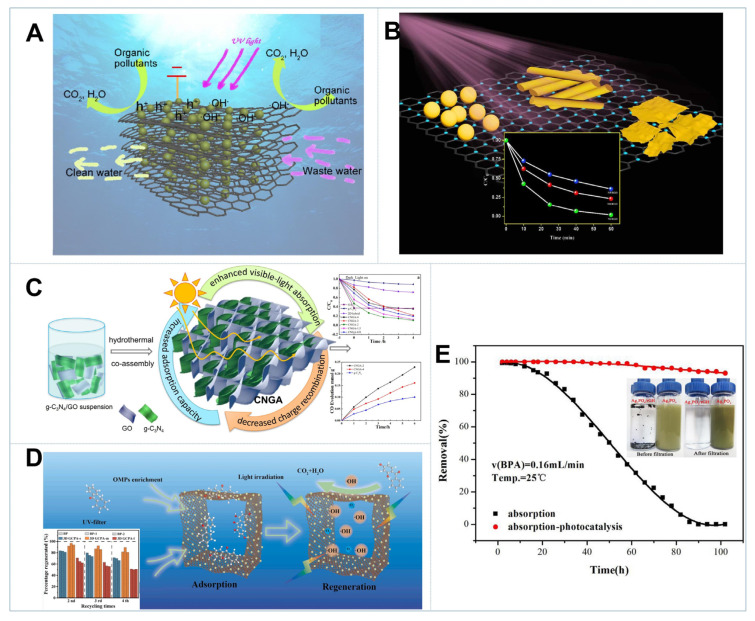
(**A**) Schematic illustration of the possible photoelectrocatalytic mechanism [136]. (**B**) Photocatalytic degradation of MB by different CdS/RGO composites [138]. (**C**) Synthesis of g-C_3_N_4_/GO aerogels (CNGA) and photocatalytic degradation of MO under visible light [139]. (**D**) 3D GCPA-m in OMPs removal and its reusability [147]. (**E**) Breakthrough curves of adsorption and adsorption-photocatalysis of Ag_3_PO_4_/rGH [150].

Zhang et al. prepared TiO_2_-Graphene Hydrogel (TGH) by a one-pot hydrothermal method, with the mass ratios of GO to TiO_2_ ranging from 4:1 to 1:5. Under UV irradiation, TGH showed much better photocatalytic activities in MB removal than TiO_2_ nanoparticles, and attained the best performance at the ratio of 1:4. The integration of TiO_2_ and 3D graphene hydrogel not only increased the SSA of the resultant hybrids, but also enabled rapid charge transport and expanded the light absorption range, which greatly enhanced pollutant adsorption and photocatalytic oxidation [135]. Moreover, TGH electrodes were also fabricated for electrocatalytic mineralization of organic pollutants. As demonstrated in Figure 5A, under the synergism of electric field and UV irradiation, numerous hydroxyl radicals and holes (h^+^) were produced on TGH surface due to the more efficient charge-separation. The removal rate of photoelectrocatalysis toward MB was 4.7 and 2 times higher than that of electrocatalysis and photocatalysis, respectively. It was also 3 times higher than that of P25, signifying the critical role of adsorption-enrichment by TGH [136]. In addition to photocatalytic oxidation, TGH displayed exceptional performance for the reduction of Cr(VI) to Cr(III) under UV irradiation. Furthermore, 100% abatement could be attained within 30 min. It also exhibited superior performance under continuous flow conditions, which was attributed to the integration of TiO_2_ and 3D graphene that promoted charge transport and separation [137].

It has been demonstrated that the dimension of semiconductors could remarkably influence the photocatalytic properties of the resultant graphene-based composites. As depicted in Figure 5B, 2D CdS nanosheet/rGO composite displayed significantly higher photocatalytic activity in MB removal over 0D nanoparticle and 1D nanorod composites under visible light irradiation. HO• radicals were generated for MB oxidation. Structural analysis revealed that strong electronic interaction was formed between CdS nanosheets and RGO. It was beneficial to transport electrons to active sites and harvest photons from solar light, thus presenting superior catalytic activity with respect to other hybrid nanomaterials [138]. As presented in Figure 5C, a 2D metal-free semiconductor with a medium band gap (2.7 eV), g-C_3_N_4_ suffered from surface aggregation and high electron-hole recombination with inefficient light absorption. The coassembly of 2D g-C_3_N_4_ and 3D GBMs could enhance the visible-light absorption and pollutant enrichment, while inhibit the radiative recombination of electron–hole pairs, which greatly improved the photocatalytic activities both in organic oxidation and heavy metal reduction [139,140]. Notably, the reaction pathways and activities would be largely dependent on the type of semiconductors. For example, BiOBr or BiOCl preferred to directly oxidize organic pollutants by the hole transfer, while TiO_2_ and g-C_3_N_4_ tended to oxidize the organics via the generation of HO• and O_2_^•−^, respectively [141,142]. Furthermore, photocatalytic performances were also dependent on the pollutant types, which exhibited different adsorption affinities to 3D GBMs and distinct reactivities toward various reactive oxygen species (ROS) [143]. Therefore, rational design and fabrication of specific 3D graphene-based photocatalysts is imperative for selective oxidation of contaminants.

In addition to the integration with metallic or nonmetallic semiconductors, Chowdhury and coworkers fabricated a series of boron-doped graphene aerogels (BGAs) via the hydrothermal assembly method. With the creation of defect regions and edge sites by B doping, it was unveiled that BGA with a moderate B doping (~2.15 at%) manifested a superior photocatalytic oxidation efficiency toward acridine orange (98%). It outperformed other BGAs with the B content ranging from 0.14 to 3.37 at% and typical photocatalysts such as Co_3_O_4_/Fe_2_O_3_, ZnO-CeO_2_, Ce-Cd and ZnO-Ag-graphene. The extraordinary performance was arising from the enhanced light absorption and inhibited electron–hole recombination [144]. N-doping, N/S codoping and B/N codoping could also render 3D GBMs with high photocatalytic activities under visible light. O_2_^•−^ was identified as the predominant ROS driving the reactions [145,146].

Considering the trace concentrations of organic micropollutants in natural water that are difficult to remove by conventional photocatalysis, the development of 3D GBMs with adsorption–photocatalysis dual functionality are highly desirable for pre-enrichment of pollutants and in situ recovery [5,116]. As discussed above, Shen et al. demonstrated that the coassembly of GO and CTFs into 3D GCPA could attain ultrahigh adsorption capacity toward 2,2′,4,4′-tetrahydroxybenzophenone (BP-2, 1862 μM g^−1^) and excellent regeneration performance under sunlight (~90% in the fourth run) (Figure 5D) [147,148,149]. Mu and coworkers fabricated 3D Ag_3_PO_4_/graphene hydrogel, which exhibited exceptionally high adsorption–photocatalytic efficiency for BPA under visible light irradiation, and complete BPA removal under the continuous flow reaction system could be maintained over 60 h (Figure 5E) [150]. So, it is a smart approach to fabricate 3D GBMs photocatalytic devices for real wastewater purification.

#### 4.2.2. Fenton-Like Advanced Oxidation

In recent years, advanced oxidation processes (AOPs) become appealing in wastewater treatment. Various oxidants including H_2_O_2_ and persulfate could be activated by metal-based or metal-free catalysts to generate highly reactive ROS for rapid degradation of organic pollutants [151]. Generally, metal-free graphene-based materials displayed poor effectiveness in H_2_O_2_ activation for pollutant abatement, but they could serve as efficient platforms for iron-containing compounds such as Fe^0^, Fe_3_O_4_ and FeOOH for the heterogeneous Fenton-like or photo-Fenton reactions. They could also act as excellent cocatalysts for the activation of H_2_O_2_ or other oxidants. Different from H_2_O_2_ activation, graphene and its derivatives showed considerable activities in persulfate activation for organic oxidation. The performance could be further improved by chemical doping, surface modification as well as metal hybridization. Moreover, 3D GBMs were more conducive to the enrichment of organic pollutants and electron transport in Fenton-like AOPs, affording ideal platforms for practical applications.

**Figure 6:**
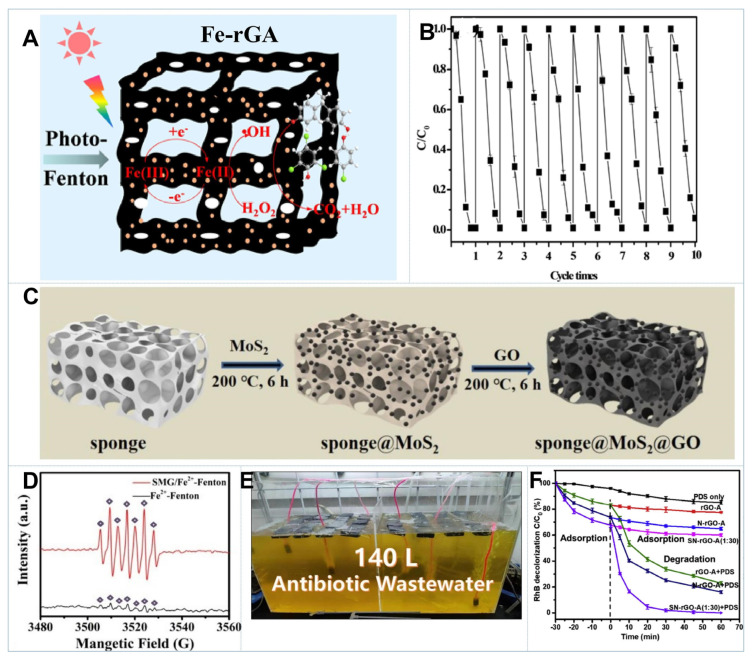
(**A**) Schematic diagram of photo-Fenton reaction over FeO(OH)-rGA [152]. (**B**) The reusability of the FeO(OH)-rGA aerogel after 10 cycles under visible light irradiation [152]. (**C**) Illustration of the fabrication processes of spong@MoS_2_@GO [158]. (**D**) EPR spectra for the detection of HO• using DMPO as the spinning trapping agent [158]. (**E**) Photograph of the 140 L degradation device [158]. (**F**) RhB degradation by PDS activation over different catalysts [165].

Zhang et al. prepared 3D reduced graphene oxide nanosheets/Fe_2_O_3_/polypyrrole hydrogels (rGSs/Fe_2_O_3_/PPy) via a one-pot reduction assembly method. The ternary hydrogels showed much better catalytic activities in H_2_O_2_ activation for MB removal than other reference samples and manifested good stability with only 2.2 wt% of Fe leaching in Fenton reaction. The outstanding performance was ascribed to the synergistic effect of strong adsorption and abundant active sites for heterogeneous catalysis [151]. Nevertheless, heterogeneous Fenton reactions also suffered from narrow pH availability (pH < 3.0), mass generation of iron cement and low utilization efficiency of H_2_O_2_ [152]. To address the abovementioned challenges, a series of macroscopic photo-Fenton catalysts including Fe_2_O_3_/GAs, CoFe_2_O_4_/GAs, FeO(OH)-rGAs and rGH/Fe-g-C_3_N_4_ were developed, and they exhibited ultrahigh and recyclable photo-Fenton activities [153,154,155,156]. For instance, Fe_2_O_3_/GAs showed good solar-driven Fenton activity for methyl orange (MO) oxidation over a broad pH range (3.5–9.0). The photo-generated electrons were prone to reduce Fe^3+^ to Fe^2+^, thus resulting in the accumulation of Fe^2+^ on the graphene surface and enhanced generation of HO• (Figure 6A). The rapid conversion of Fe^3+^/Fe^2+^ would also significantly inhibit the generation of Fe(OH)_3_, so Fe_2_O_3_/GAs could maintain their high activities (Figure 6B) even at high pH conditions [157,158].

It has been demonstrated that the addition of reductants such as MoS_2_, WS_2_, boron and black-red phosphorus could accelerate the reduction of Fe^3+^ and improve the cocatalytic performance in Fenton and Fenton-like reactions [159]. In order to effectively promote H_2_O_2_ activation and be easily recycled, Zhu et al. fabricated the 3D sponge@MoS_2_@GO (SMG) by dipping a blank melamine sponge into MoS_2_ and GO alcohol solution followed by heat treatment at 200 °C, as shown in Figure 6C [158]. EPR spectra (Figure 6D) revealed that the seven-line signals referring to HO• were significantly strengthened in the SMG/Fe^2+^-H_2_O_2_ system than those in conventional Fenton system, indicating that SMG could greatly enhance HO• production for pollutant abatement. Compared with MoS_2_ powder catalyst, 3D SMG had integrated morphology, interconnected pores, hydrophobicity and larger SSA. It was more conducive to the circulation of Fe^3+^/Fe^2+^, the enrichment of organic pollutants and the recycling of catalyst. Moreover, SMG also displayed excellent cocatalytic performance in the Fe^2+^-PMS system for pollutant oxidation with fewer toxic by-products generated. A pilot-scale experiment (Figure 6E) unveiled that SMG/Fe^2+^-PMS system could attain 97.87% removal of 120 mg L^−1^ antibiotic wastewater under 16 days of continuous reaction. The operating cost of treating a ton of wastewater was only USD 0.33, implying huge potential for industrial applications [158].

In contrast to traditional Fenton reactions that primarily relied on radical oxidation, the cleavage of peroxide O-O bond in peroxymonosulfate (PMS) and persulfate (PDS) would lead to the generation of either radical (SO_4_^•−^, HO•, O_2_^•−^) or nonradical ROS (carbon-activated persulfate complexes, ^1^O_2_) [160]. Notably, radical-based AOP systems benefited from high redox potentials for sufficient mineralization of pollutants, but the oxidation efficiency would be greatly impacted by the coexisting inorganic anions and natural organic matters (NOMs) in complicated water matrices. Nevertheless, nonradical-based AOP systems displayed high selectivity toward electron-rich organic pollutants and maintained their excellent efficiencies in a wide pH range despite the copresence of NOMs and inorganic ions. Their redox potentials were also tunable to be regulated by surface properties and composition of the catalysts [160].

Substitutional doping with heteroatoms such as N, S, B or P into the carbon lattice has been confirmed to be an effective strategy to enhance the catalytic activity [161,162,163]. Our group constructed 3D N-doped graphene aerogels (NGA) by facial hydrothermal assembly of GO and melamine. NGA showed excellent catalytic activity in PMS activation for ibuprofen oxidation, which attained 8- and 44-fold enhancement in reaction rate over N-dope reduced graphene oxide (NrGO) and graphene aerogel (GA), respectively. By regulating the GO concentrations, the mass ratios of GO to melamine and thermal treatment temperatures, a series of NGA samples have been fabricated. Structure–property analysis revealed that graphitic N was the intrinsic catalytic center for PMS-based oxidation, while the 3D porous network was favorable for mass transport, which synergistically promoted pollutant abatement [13]. These findings were consistent with Ren’s study, which also demonstrated that graphitic N doping could enhance PMS adsorption as well as elevate the potential of derived nonradical species and boost the pollutant oxidation via electron transfer process (ETP) [18]. For nonradical ETP oxidation, the redox potential depends on the persulfate adsorption amounts on the carbon surface. A higher adsorption amount would lead to a greater redox potential and subsequently faster reaction kinetics [164]. Notably, as presented in Figure 6F, N-doping on carbon substrate could barely promote PDS activation for organic abatement, but it was found that N and S-codoped graphene aerogel (SN-rGO-A) exhibited considerably enhanced performance in PDS activation for MO removal. It should be attributed to the significant changes in the surface charge distribution as reflected by DFT calculation [165].

As it is known, the reusability of metal-free catalysts is mediocre due to the low mineralization rate and coverage of intermediates. The incorporation of metal/metal oxide could promote radical generation and accelerate organic mineralization, thereby improving the stability of hybrid catalysts and maintaining the intrinsic selectivity [166]. A 3D graphene framework is a perfect platform for stabilizing transition metals [160]. Yi and coworkers prepared 3D CoOOH/GO hydrogel and filled it into the flowing column to achieve automatic purification of wastewater. The 3D hydrogel could effectively activate PMS to produce SO_4_^•−^ and ^1^O_2_ for abating a series pollutants including phenol, rhodamine B, bisphenol A, norfloxacin, tetracycline hydrocholoride and p-chlorophenol [167]. The presence of GO hydrogel could remarkably inhibit Co^x+^ leaching and improve the recyclability of the catalyst, which paved a novel way for the wastewater treatment. In a recent work by our group, the integration of MnO_2_ nanosheets and 3D N-doped graphene aerogel (S-MnO_2_/NGA) demonstrated an exceptional catalytic activity in PMS activation for ibuprofen oxidation over NGA and S-MnO_2_. The intimate interactions between 2D MnO_2_ nanosheet and 3D porous framework were advantageous for simultaneously improving charge transport and shortening the diffusion route of pollutants to promote mass transfer, which manifested a wide adaptability in catalytic oxidation of various organic pollutants irrespective of solution pH and coexisting anions [14]. Beyond that, numerous novel metal-based 3D GBMs such as Co_3_O_4_/NGA, CoFe_2_O_4_/NGA and Fe_2_O_3_/NGA have been developed for high-efficient catalytic activation of persulfate for wastewater remediation. Further investigation of 3D graphene-based single-atom-catalysts (SACs) are highly desirable for PS-AOPs in environmental applications [151,165,168,169,170].

### 4.3. Capacitive Deionization (CDI)

**Figure 7:**
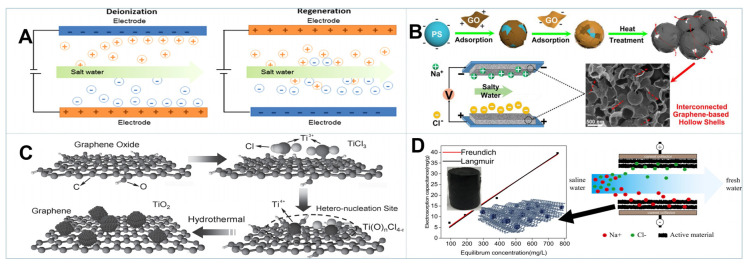
(**A**) Schematic illustration of CDI device [64]. (**B**) Illustration of the preparation of 3D graphene-based hollow shells for CDI applications [65]. (**C**) Formation mechanism of GA/TiO_2_ hybrids [175]. (**D**) The adsorption model schematic of MOF/HG composite [179].

Capacitive deionization (CDI) is an emerging electrochemically controlled technique which could achieve energy efficient and cost-effective water desalination based on electric double-layer capacitors (EDLCs) [171]. As presented in Figure 7A, when an external voltage is applied, charged ions can form EDLCs by adhering to the surface or into the pores of porous electrodes. When the charge is withdrawn or reversed, the ions can be released from the electrodes [171]. So, the electrode materials are crucial in the CDI process. Carbon materials such as carbon nanotubes, activated carbon and graphene have been frequently employed as electrode materials for CDI due to their high chemical stability, tunable porous structure as well as good electrical conductivity. Having benefited from the high specific surface area of 3D GBMs, fast ion/electron transfer and high electro sorption capacity could be triggered to achieve a better CDI performance. Moreover, rational fabrication of 3D GBMs via the optimization of porous structure and hybridization with nanomaterials could further improve the desalination capability [171,172].

Template and template-free methods were generally applied to modulate the porous structure of 3D GBMs. As shown in Figure 7B, Wang and coworkers fabricated 3D graphene with abundant micro-/mesopores by utilizing polystyrene (PS) as the templates, and followed this with chemical etching. Due to the high conductivity and specific surface area, the as-obtained 3D graphene manifested a high salt electrosorption capacity of 14.4 mg g^−1^ and good regeneration performance [173]. Kong et al. prepared a 3D graphene hydrogel with ample in-plane pores by H_2_O_2_ etching. The in-plane pores allowed the barrier-free access to the interior surface of the electrode, which greatly boosted the CDI performance with a desalination capacity of 26.8 mg g^−1^ [65]. Moreover, more micro-/mesopores could be introduced into the 3D GBMs by chemical activation using KOH or H_3_PO_4_ as the activators. The as-obtained 3D graphene had a hierarchical porous structure and ultrahigh SSA up to 3513 m^2^ g^−1^, displaying extensive prospect for CDI applications [174].

Due to the high pseudocapacitance of metal oxides, the incorporation of metal oxide into graphene aerogel could integrate the superiorities of high specific capacitance and fast mass transfer, thereby enhancing the desalination efficiency [171,172]. As shown in Figure 7C, Yin et al. proposed a well-defined 3D graphene aerogel/metal oxide (GA/MO) hybrid using GO and low-valence metal ions as the precursors [175]. It was unveiled that GA/TiO_2_ hybrid displayed high electron sorption capacity (25.0 mg g^−1^) and reaction rate with equilibrium attained within 200 s. It also outperformed other hybrid materials, including GA/Fe_3_O_4_, GA/CeO_2_ and GA/Mn_3_O_4_ [175,176]. Moreover, the structural features of MO such as morphology, crystal phase and size might result in distinguished performance of electrode, which deserves further investigation.

In addition to MO, the introduction of conducing polymers, MOFs and nanocarbons could also increase the SSA as well as electrical conductivity of 3D GBMs, which pave new ways to achieve high CDI performance [177,178]. As presented in Figure 7D, Feng and coworkers prepared 3D MOF/holey graphene (HG) composites as electrode materials for desalination applications. The MOF/HG-2 with an MOF-to-HG ratio of 2:1 exhibited an optimum electrosorption capacity of 39.6 mg g^−1^ in 800 mg·L^−1^ NaCl solution at 1.4 V [179]. Furthermore, the 3D coassembly of CNTs with graphene could not only optimize the porous structure that facilitate ion diffusion, but also form the “plane-to-line” conducive channels that increase ion conduction, thus manifesting better performances for CDI applications [180].

## 5. Conclusions and Outlook

Over the past decade, 3D GBMs have attracted substantial research interests worldwide due to their outstanding physical and chemical properties in terms of controllable structures and easy manipulation, affording great potential for water treatment applications. This review covers the structural manipulation of 3D GBMs and different applications in water purification, which attempts to provide a deep understanding of the multiple roles of 3D GBMs for improved pollutant abatement. Despite considerable research progress, some concerns should also not be ignored. First, the common fabricated categories such as the template-based CVD method and hydrothermal assembly followed by freeze-drying are time-consuming and costly; cost-effective production of high-quality 3D GBMs for water treatment remains challenging. Second, oriented porous structures endow 3D GBMs with fascinating properties; the synergies between oriented 3D framework and diverse active components are barely explored. Third, there are substantial NOMs, anions and microorganisms in natural water; the effects of these substances on the adsorption or catalytic performance of 3D GBMs for pollutant removal are still ambiguous. In summary, the fabrication of low-cost, highly reactive and stable 3D GBMs will open up practical applications in water purification in the future.

## Figures and Tables

**Table 2:** A Summary of Adsorption Capacities and Mechanisms of Organic Pollutants and Heavy Metals onto 3D GBMs.

Samples	Pollutants	pH	Adsorption Capacity (mg/g)	Adsorption Mechanisms	Ref.
3D GO	MV	7.0	401	Electrostatic attraction, π-π interaction	[101]
	MB	7.0	343		
3D G-CNT	Oxytetracycline	7.0	1729	π-π interaction, n-π EDA interaction	
	Diethyl phthalate	7.0	680		[98]
	Methylene blue	7.0	685		
	Cd^2+^	7.0	235	Ion exchange, surface complexation	
β-CD/GO aerogel	Methyl orange	-	167.0	Electrostatic attraction, H-bonding, π-π interaction	[102]
	Acid red	-	87.2		
	Methylene blue	-	439.0		
	Rhodamine B	-	388.0		
3D GO-chitosan	U(VI)	3.5	200.0	Electrostatic attraction, ion-exchange, surface complexation	[103]
		5.0	319.9		
		8.3	384.6		
Graphene-MoS_2_ aerogel	Hg^2+^	5.0	970	Electrostatic attraction	[104]
	Pb^2+^	5.0	1245		
Graphene/δ-MnO_2_ aerogel	Pb^2+^	6.0	643.6	Electrostatic attraction, ion exchange, surface complexation	[105]
	Cd^2+^	6.0	250.3		
	Cu^2+^	6.0	228.5		
NH_2_-Graphene sponge	Cr^6+^	4.1	166.5	Electrostatic attraction	[106]
Lignosulfonate-Graphene hydrogel	Pb^2+^	5.0	1210.0	Electrostatic attraction, ion exchange, surface complexation	[107]

## Data Availability

Not applicable.
